# Dietary capsaicin and its anti-obesity potency: from mechanism to clinical implications

**DOI:** 10.1042/BSR20170286

**Published:** 2017-05-11

**Authors:** Jia Zheng, Sheng Zheng, Qianyun Feng, Qian Zhang, Xinhua Xiao

**Affiliations:** 1 Department of Endocrinology, Key Laboratory of Endocrinology, Ministry of Health, Peking Union Medical College Hospital, Diabetes Research Center of Chinese Academy of Medical Sciences & Peking Union Medical College, Beijing 100730, China; 2 Graduate School, Tianjin University of Traditional Chinese Medicine, Tianjin 300193, China

**Keywords:** adipogenesis, appetite, brown adipose tissue, Capsaicin, obesity, TRPV1

## Abstract

Obesity is a growing public health problem, which has now been considered as a pandemic non-communicable disease. However, the efficacy of several approaches for weight loss is limited and variable. Thus, alternative anti-obesity treatments are urgently warranted, which should be effective, safe, and widely available. Active compounds isolated from herbs are similar with the practice of Traditional Chinese Medicine, which has a holistic approach that can target to several organs and tissues in the whole body. Capsaicin, a major active compound from chili peppers, has been clearly demonstrated for its numerous beneficial roles in health. In this review, we will focus on the less highlighted aspect, in particular how dietary chili peppers and capsaicin consumption reduce body weight and its potential mechanisms of its anti-obesity effects. With the widespread pandemic of overweight and obesity, the development of more strategies for the treatment of obesity is urgent. Therefore, a better understanding of the role and mechanism of dietary capsaicin consumption and metabolic health can provide critical implications for the early prevention and treatment of obesity.

## Introduction

The epidemic of obesity is a growing public health problem. The incidence of obesity has more than doubled since 1980, and has now reached worldwide epidemic status [1]. In 2014, the World Health Organization (WHO) estimated that 39% of the human adult population with 1.9 billion people were affected with overweight (body mass index (BMI) ≥25 kg/m^2^), and that obesity (BMI ≥30 kg/m^2^) affected approximately 13% with 600 million people [2,3]. Obesity is a serious risk factor as it is associated with chronic inflammation and metabolic syndrome [4], a cluster of morbidities that includes hypertension, hyperlipidemia, and Type 2 diabetes mellitus (T2DM) [5]. It can increase the risks of developing serious health problems, such as cardiovascular diseases, chronic kidney disease, and stroke [6,7]. Moreover, obese patients are more prone to contract several forms of cancer with reduced chances of survival [8]. Of particular concern is the incidence of overweight and obesity in children, with an estimated one-third of children and adolescents affected in the United States and over 41 million children are overweight before reaching puberty [2]. As such, obesity and its related diseases yield enormous tolls at individual, public health, and economic levels. In addition, genome-wide association studies (GWAS) have revealed compelling genetic signals influencing obesity risk, and genetic polymorphism plays a major role in determining obesity [9]. An updated randomized controlled trial indicated the higher body weight and waist circumference reductions in risk carriers than in non-risk carriers of the fat mass and obesity-associated (FTO) gene across different levels of personalized nutrition [10]. These data signify that the interventions should be personalized and vary with each individual [11]. Thus, the development of novel and personalized strategies for the early prevention and treatment of overweight and obesity is warranted.

## Limitations in anti-obesity approaches

It has clearly established that weight loss will significantly diminish the complications of obesity [12]. Emerging human epidemiology studies indicated that reducing body weight, with weight loss of at least 5%, has long-term benefits on metabolic health and reduces the risks of developing insulin resistance, T2DM, and cardiovascular diseases [13]. However, weight loss is difficult and the obese individuals are struggling to achieve it, and the efficacy of several approaches for weight loss is limited and variable [14,15]. Firstly, it is widely accepted that a combination of physical exercise and low-calorie diet is the best approach to prevent and treat obesity. However, this strategy is difficult to implement and its compliance is poor. Gupta et al. [16] aimed to explore treatment satisfaction associated with different weight loss methods among patients with obesity. It showed that using self-modification weight loss techniques such as diet, exercise, and weight loss supplements has lowest treatment satisfaction, compared with gastric bypass and gastric banding, and prescription medication [16]. In addition, physical exercise and diet intervention also yield enormous tolls at economic level. It reported that retail sales of weight loss supplements were estimated to be more than $1.3 billion in 2001 in United States [17]. Thus, cheap, easily available therapies and supplements are urgently needed. The second approach is pharmaceutical drugs, such as orlistat, a potent and specific inhibitor of intestinal lipases. It can reduce body weight with an average weight loss of 3% during 1 year period [13]. However, its efficacy is variable and it can lead to gastrointestinal adverse effects, liver failure, and acute kidney injury [18]. Other anti-obesity drugs, such as rimonabant, fenfluramine, and sibutramine, have been withdrawn from the market due to severe adverse effects, including increased cardiovascular risks, mood disorders, and even suicidal susceptibility [14]. Thirdly, anti-diabetic agents, such as glucagon-like peptide 1 (GLP-1) analog, liraglutide has been shown its potential anti-obesity efficacy [19]. But it needs to be injected subcutaneously daily. Moreover, the weight loss is limited and it can increase the risk of pancreatitis [20]. Compared with aforementioned anti-obesity drugs, bariatric surgery such as Roux-en-Y gastric bypass or sleeve gastrectomy is more effective. However, it is physically invasive, relatively expensive, and its long-term effect is unclear [21]. Therefore, alternative anti-obesity treatments are urgently warranted, which should be effective, safe, and widely available [15].

## An overview of chili peppers and capsaicin

**Figure 1:**
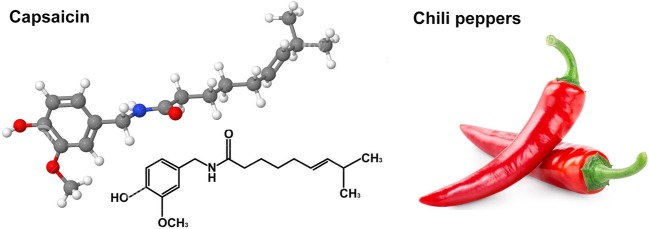
The molecular structure of capsaicin isolated from chili peppers

Chili pepper is generally used as a flavoring spice and is prominent in diets of various communities and cultures worldwide since 7000BC, with a long history of flavoring, coloring, preserving food as well as medication [26]. In chili pepper, more than 200 active constituents have been identified and some of its active constituents play multiple roles in the whole body [27]. Capsaicin, as a major active compound from chili pepper, has been established for its numerous beneficial roles in the human organism, including the treatment of pain inflammation, rheumatoid arthritis [28], and vasomotor rhinitis (Figure 1) [29]. Furthermore, capsaicin has proven an effective anti-cancer agent. Several preclinical studies showed that capsaicin could suppress various human neoplasia by generating reactive oxygen species and increasing apoptosis [30,31]. Finally, capsaicin demonstrated significant antioxidant properties, and it was postulated that this compound has important implications in the prevention or treatment of neurodegenerative diseases such as Alzheimer’s disease [32]. In addition to capsaicin as anti-obesity compounds, other types of natural products also have shown to be considered as anti-obesity compounds. Celastrol (from roots of the thunder god vine) can reduce appetite and food intake in mice that are fed a high-fat diet [33]. Stilbenoid resveratrol (from grapes and red wine), genistein (an isoflavone from soy), glycyrrhizin (from liquorice), quercetin, ethanolic extract (from ginseng roots), and green tea extract (including camellia sinensis, catechin, caffeine, and epigallocatechin gallate) play a role in adipogenesis inhibition, thus may have anti-obesity potency [15]. In this review, we will focus on the less highlighted aspect, in particular how dietary chili peppers and capsaicin consumption reduce body weight and the potential mechanisms of its anti-obesity effects. Figure 1 shows the molecular structure of capsaicin isolated from chili peppers.

## Clinical studies of the weight-loss effects of capsaicin

### Weight-loss effects of capsaicin on lipid oxidation and energy expenditure

Epidemiological data revealed that the consumption of foods containing capsaicin was associated with a lower prevalence of obesity [34]. In one double-blind, randomized, placebo-controlled trial, it indicated that treatment of overweight or obese subjects with 6 mg/day capsinoid for 12 weeks was associated with abdominal fat loss measured by dual energy X-ray absorptiometry. Body weight was decreased as 0.9 and 0.5 kg in the capsinoid and placebo groups respectively. Moreover, none of the patients developed any adverse events (Table 1) [35]. Lejeune et al. [36] aimed to investigate whether capsaicin assists weight maintenance by limiting weight regain after weight loss of 5% to 10%. The results showed that capsaicin treatment caused sustained fat oxidation during weight maintenance compared with placebo (Table 1) [36]. Increase in the oxygen consumption (VO_2_) and body temperature reflecting increased energy expenditure, thus play critical role in weight loss. Fat oxidation was reported to be sustained together with elevation of the resting energy expenditure, and enhanced fat oxidation may contribute to increased energy expenditure. In another randomized double-blind study, it indicated that subjects between 30 and 65 years old with a BMI >23 kg/m^2^ treated with capsinoid (10 mg/kg per day) for 4 weeks safely and body weight tended to decrease during the 2- to 4-week period, with increased VO_2_, resting energy expenditure, and fat oxidation significantly (Table 1) [37]. Enhanced lipid oxidation and increased energy expenditure are potentially beneficial for weight management [38].

**Table 1:** Clinical studies of the weight-loss effects of capsaicin

Treatments	Year	Country	Study design	Subjects included	Baseline BMI	Sample size	Age (years)	Outcomes	Adverse events	Potential mechanism	Reference
Capsinoids (6 mg per day for 12 weeks)	2009	U.S.A.	Double-blind, randomized, placebo-controlled trial	Overweight individuals	30.6 ± 2.4	*N*=80	42 ± 8	Body weight decreased 0.92 kg; abdominal fat decreased 1.11%	None	Increase in fat oxidation and genetic polymorphisms	Snitker et al. [35]
Red pepper (capsaicin 10 g single meal)	1999	Canada	Prospective study	Healthy individuals	25.3 ± 4.7	*N*=23	25.8 ± 2.8	Decreased appetite	None	Increase in sympathetic nervous system activity	Yoshioka et al. [40]
Capsinoids (10 mg/kg per day for 4 weeks)	2007	Japan	Double-blind, randomized, placebo-controlled trial	Men and postmenopausal women	>23	*N*=48	30–65	Body weight tended to decrease during the 2- to 4-week period	None	Increased VO_2_, energy expenditure, and fat oxidation	Inoue et al. [37]
Capsaicin (135 mg per day for 3 months)	2003	Netherlands	Randomized double-blind placebo-controlled study	Moderately overweight subjects	29.3 ± 2.5	*N*=140	18–60	Significant increase in resting energy expenditure	None	More sustained fat oxidation	Lejeune et al. [36]
Capsinoids (9 mg per day for 8 weeks)	2016	Japan	Randomized double-blind placebo-controlled study	College students	21.4 ± 1.8	*N*=20	20.7 ± 1.2	Increased brown adipose tissue (BAT) density	None	Increased BAT activity	Nirengi et al. [43]

### Weight-loss effects of capsaicin on appetite and brown adipose tissue

Dietary red pepper can suppress energy intake and modify macronutrient intake through appetite and satiety regulation [39]. One prospective study aimed to investigate the effects of capsaicin on feeding behavior and energy intake. It indicated that the addition of red pepper to the breakfast significantly decreased protein and fat intakes at lunch time, and the addition of red pepper to the appetizer significantly reduced the cumulative ad libitum energy and carbohydrate intakes during the rest of the lunch. These effects might be related to an increase in sympathetic nervous system activity (Table 1) [40]. BAT is known to play a critical role in cold-induced non-shivering thermogenesis to maintain body temperature, and it is expected to be a therapeutic target for obesity and related metabolic disorders in humans [41]. It showed chili pepper affects energy expenditure by triggering the BAT in the same way as low temperature does, leading to increased energy expenditure via non-shivering thermogenesis [42]. One recent clinical study showed that 9 mg of capsinoid for 8 weeks could increase BAT activity and increase thermogenesis in healthy subjects (Table 1) [43]. The results suggest that dietary capsaicin consumption could have a beneficial effect for weight management, by reducing energy intake and activation of BAT activity. The summary of the clinical studies about the weight-loss effects of capsaicin was shown Table 1.

## Preclinical studies about anti-obesity effects of capsaicin and its potential mechanisms

### Capsaicin and TRPV1 activation

Numerous epidemiology studies and animal studies indicated that capsaicin, as a transient receptor potential vanilloid 1 (TRPV1) agonist, may represent a potential strategy to treat obesity. Although it is well accepted that much of the effect is caused by stimulation of the TRPV1 receptor, the mechanism of action is not presently fully understood. Increasing evidence indicates that TRPV1 plays a critical role in the regulation of metabolic health for the whole body, including body weight, glucose and lipid metabolism, and cardiovascular system [44,45]. TRPV1 was deemed as a potential target for the prevention of obesity due to its effect on energy metabolism and balance [46,47]. Activation of TRPV1 by capsaicin can attenuate abnormal glucose homeostasis by stimulating insulin secretion and increasing GLP-1 levels (Table 2) [48,49]. Furthermore, capsaicin also plays its role in a receptor-independent manner. It reported that capsaicin was associated with nuclear factor-κB (NF-κB) inactivation and peroxisome proliferator-activated receptor-γ (PPARγ) activation, and then it could modulate the adipocyte function of adipose tissues in obese mouse and suppressed the inflammatory responses of adipose tissue macrophages, which are independent on TRPV1 [50]. Additionally, TRPV1 can play a critical role in cell proliferation and cancer. It showed that TRPV1 implicated as a regulator of growth factor signaling in the intestinal epithelium, which could subsequent suppress intestinal tumorgenesis [51].

**Table 2:** Preclinical studies about anti-obesity effects of capsaicin

Treatments	Species	Duration	Metabolic disorders	Potential mechanism	Reference
0–250 μmol/l capsaicin	3T3-L1 preadipocytes and adipocytes	24–72 h	Decreased the amount of intracellular triglycerides, GPDH activity	Inhibited the expression of PPARγ, C/EBP-α, and leptin	Hsu et al. [52]
			Induced apoptosis	Induced up-regulation of adiponectin at the protein level	
			Inhibited adipogenesis		
1 μmol/l capsaicin	3T3-L1 preadipocytes	3–8 days	Prevented the adipogenesis	Increased intracellular calcium	Zhang et al. [53]
0.015% capsaicin	Male C57BL/6 mice	10 weeks	Decreased triglyceride levels	Decreased TRPV-1 expression in adipose tissue	Kang et al. [54]
			Lowered fasting glucose, insulin, and leptin levels	Increased mRNA/protein of adiponectin in the adipose tissue	
				Increased PPARα/PGC-1α mRNA in the liver	
10 mg/kg body weight capsaicin	Std ddY mice	2 weeks	Lower body weight	Increased oxygen consumption	Ohnuki et al. [55]
			Markedly suppressed body fat accumulation	Stimulated the secretion of adrenalin	
			Decreased triglyceride levels		
0.3% capsinoids	C57BL/6J mice	8 weeks	Suppressed body weight gain under the HFD	Increased energy expenditure	Saito et al. [57], Ohyama et al. [58]
			Decreased plasma cholesterol level	Activation of fat oxidation in skeletal muscle	
			Prevented diet-induced liver steatosis	Activation lipolysis in BAT	
				Increased cAMP levels and PKA activity in BAT	
0.003%, 0.01%, and 0.03% capsaicin	wild-type and TRPV1−/− mice	16 weeks	Promoted weight loss	Increased the expression of UCP-1, BMP8b, SIRT-1, PGC-1α, and PRDM-16 in BAT	Baskaran et al. [59]
			Enhanced the respiratory exchange ratio	Increased the phosphorylation of SIRT-1	
			Countered hypercholesterolemia		
0.01% capsaicin	wild-type and TRPV1−/− mice	26 weeks	Countered obesity	Promoted SIRT-1 expression	Baskaran et al. [60]
			Browning of WAT	Increased the expression of PGC-1α	
				Facilitated PPARγ–PRDM-16 interaction	
0.01% capsaicin	wild-type and TRPV1−/− mice	24 weeks	Ameliorated abnormal glucose homeostasis	Activation of TRPV1-mediated GLP-1 secretion in the intestinal cells	Wang et al. [49]
			Increased GLP-1 levels in the plasma and ileum		
640 μmol/L, 2 ml/kg capsaicin	Sprague-Dawley rats	15 min	Increased superior mesenteric artery blood flow	Induced a dichotomous pattern of blood flow changes	Leung et al. [68]
			Reduction in hydrogen gas clearance		
0.01% capsaicin	C57BL/6J male mice	9 weeks	Reduced weight gain	Modest modulation of the gut microbiota	Shen et al. [69]
			Improved glucose tolerance	Up-regulated the expression of *Muc2* and antimicrobial protein gene *Reg3g* in the intestine	

BMP8b, bone morphogenetic protein-8b; cAMP, cyclic adenosine monophosphate; C/EBP-α, CCAAT-enhancer-binding protein-α; GPDH, glycerol-3-phosphate dehydrogenase; Muc2, mucin 2 gene; PGC1-α, PPARγ co-activator 1-α; PKA, protein kinase A; PRDM-16, positive regulatory domain containing 16; Reg3g, regenerating islet-derived protein 3γ; SIRT-1, sirtuin-1; UCP-1, uncoupling protein 1; WAT, white adipose tissue.

**Figure 2:**
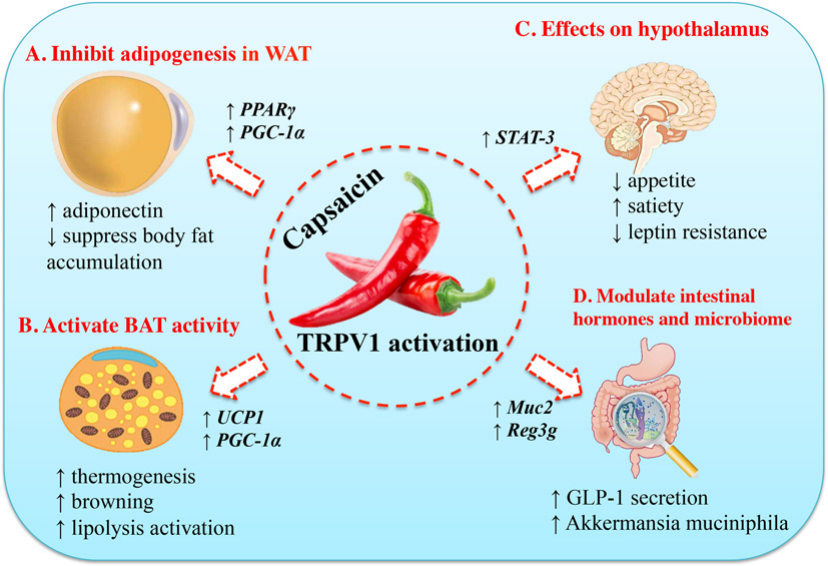
Molecular mechanisms of the anti-obesity effects of capsaicin (**A**) Capsaicin can inhibit adipogenesis in preadipocyte and adipocyte by up-regulating the expression of PPARγ and UCP-1. Thus, it will stimulate adiponectin secretion and increase body fat accumulation. (**B**) Capsaicin can activate BAT activity, accompanied by increased expression of UCP-1 and PGC-1α. (**C**) Capsaicin can suppress appetite, increase satiety, and ameliorate insulin resistance. (**D**) Capsaicin can modulate its function in gastrointestinal tract and gut microbiome, including stimulation of GLP-1 secretion and increase in population of the gut bacterium *Akkermansia muciniphila*. STAT-3, signal transducer and activator of transcription-3.

The potential mechanisms underlying the anti-obesity effects of capsaicin include: (1) increase lipid oxidation and inhibit adipogenesis; (2) activate BAT activity and induce thermogenesis; (3) suppress appetite and increase satiety regulated by neuronal circuits in the hypothalamus; (4) modulate the function of gastrointestinal tract and gut microbiome. The molecular mechanisms of the anti-obesity effects of capsaicin were summarized in Figure 2. In addition, we further collected most preclinical studies, including *in vitro* studies and rodent experiments about the anti-obesity effects of capsaicin (Table 2).

### Capsaicin and its role in adipogenesis

Adipogenesis is the critical and original process of fatty adipose accumulation. It suggested that decreased preadipocyte differentiation, proliferation, and lipogenesis have the potential to reduce obesity. Hsu et al. [52] demonstrated that capsaicin inhibited the expression of PPARγ, C/EBP-α, and leptin, but induced up-regulation of adiponectin at the protein level. Thus, it efficiently induced apoptosis and inhibited adipogenesis in 3T3-L1 preadipocytes and adipocytes *in vitro* (Table 2, Figure 2) [52]. Zhang et al. [53] found that capsaicin treatment prevented adipogenesis of 3T3-L1-preadipocytes *in vitro*, with increased intracellular calcium (Figure 2). Male C57BL/6 obese mice fed a high-fat diet for 10 weeks received a supplement of 0.015% capsaicin showed decreased fasting glucose, insulin, leptin concentrations, and markedly improved glucose intolerance in obese mice, accompanied with decreased TRPV-1 expression in adipose tissue, increased adiponectin expression in the adipose tissue, and increased peroxisome proliferator-activated receptor-α (PPARα) and PGC-1α expression in the liver (Table 2, Figure 2) [54]. Ohnuki et al. [55] demonstrated that mice treated with 10 mg/kg body weight capsaicin could markedly suppressed body fat accumulation and promoted energy metabolism (Table 2). Hence, these studies supported that capsaicin could decrease adipogenensis and regulate genes function related with lipid metabolism, and then it can has the potential to lose weight.

### Capsaicin and its role in brown adipose tissue

BAT is the main site of adaptive thermogenesis and experimental studies have associated BAT activity with protection against obesity and metabolic diseases [56]. A review illustrated that the activity of BAT can be activated and recruited not only by cold exposure but also by various food ingredients, such as capsaicin in chili pepper (Table 2) [57]. Capsinoids supplementation with exercise in C57BL/6J mice additively decreased body weight gain and fat accumulation, and increased whole body energy expenditure compared with exercise alone. The underlying mechanisms may be associated with increased energy expenditure, lipolysis activation in BAT, and increased cyclic adenosine monophosphate (cAMP) levels and PKA activity in BAT (Table 2, Figure 2) [58]. One up-to-date rodent experiment showed that capsaicin could counter the detrimental effects of high-fat diet, including glucose intolerance, hypercholesterolemia, and suppressed activity in BAT. These effects were mainly by increasing the expression of metabolically important thermogenic genes, including UCP-1, BMP8b, SIRT-1, PGC-1α, and positive regulatory domain containing zinc finger protein 16 (PRDM-16) in BAT. Furthermore, capsaicin supplementation, post high-fat diet, promoted weight loss and enhanced the respiratory exchange ratio. All these data suggested that capsaicin is a novel strategy to counter diet-induced obesity by enhancing metabolism and energy expenditure (Table 2, Figure 2) [59]. Baskaran et al. showed that activation of TRPV1 channels by dietary capsaicin triggered browning of adipose tissue to counteract obesity (Table 2) [60]. Collectively, these observations provide evidence that capsaicin can activate and recruit BAT, which would be a promising strategy to counter obesity.

### Capsaicin and its role in appetite and satiety

Energy balance requires an ability of the brain to detect the status of energy stores and match energy intake with expenditure, and energy homeostasis is mainly controlled by neuronal circuits in the hypothalamus [61]. Hypothalamic endoplasmic reticulum stress occurs in individuals with obesity and is thought to induce low levels of leptin receptor signaling and play a central role in development of leptin resistance [62]. The adipose tissue-derived hormone leptin acts via its receptor in the brain to regulate energy balance and neuroendocrine function. Leptin resistance is a pathological condition, which means the lack of appetite reduction in response to leptin and the body fails to adequately respond to it [63]. Lee et al. [64] found that TRPV1 had a major role in regulating glucose metabolism and hypothalamic leptin’s effects in obesity, with hypothalamic STAT-3 activity blunted in the TRPV1 knockout mice (Figure 2). Addition of dietary capsaicin has been shown to increase satiety and it indicated that capsaicin increased sensation of fullness in energy balance, and decreased desire to eat after dinner in negative energy balance [65]. Although the studies about capsaicin and its role in appetite is limited, it inspired us that neuronal circuits in the hypothalamus may be a pivotal target of capsaicin.

### Capsaicin and its role in gastrointestinal tract and gut microbiome

Capsaicin is passively absorbed in the stomach with greater than 80% efficiency and upper portion of the small intestine [66]. Thus, it may activate local TRPV1 channels in gastrointestinal tract to initiate a series of physiological effects. Dietary capsaicin consumption triggered the intestinal mucosal afferent nerves and increased intestinal blood flow [67]. Acute single administration of 640 μmol/l capsaicin into the duodenal lumen in anesthetized rats significantly increases superior mesenteric artery blood flow (Table 2) [68]. In addition, it showed that dietary capsaicin ameliorated abnormal glucose homeostasis and increased GLP-1 levels in the plasma and ileum through the activation of TRPV1-mediated GLP-1 secretion in the intestinal cells and tissues (Table 2, Figure 2) [49]. Recent study demonstrated that anti-obesity effect of capsaicin in mice fed with high-fat diet was associated with an increase in population of the gut bacterium *Akkermansia muciniphila*. Further studies found that capsaicin directly up-regulated the expression of Muc2 and antimicrobial protein gene Reg3g in the intestine (Table 2, Figure 2) [69]. These data suggested that the anti-obesity effect of capsaicin is associated with a modest modulation of the function in gastrointestinal tract and gut microbiome.

## Conclusions

In summary, capsaicin plays a critical role in human and has multiple benefits for metabolic health, especially for weight loss in obese individuals. It is well accepted that the potential application of active compounds isolated from herbs are similar with the practice of traditional Chinese medicine, which has a holistic approach that can target to different organs and tissues in the whole body. More importantly, no adverse effects with capsaicin were observed in most studies. Thus, chili peppers and capsaicin are safely and easily applicable to our daily life. Considering that chili peppers have been a vital part of culinary cultures worldwide and have a long history of use for flavoring, so it is more feasible to be utilized to treat overweight and obesity, compare with medications or other interventions with certain side effects. Dietary chili peppers supplementation or to be food additives, with ideal dosage may be tentative methods for capsaicin to play its protective roles in metabolic health. With the widespread pandemic of overweight and obesity, the development of more strategies for the treatment of obesity is urgent. Therefore, a better understanding of the role and mechanism of dietary capsaicin consumption and metabolic health can provide critical implications for the early prevention and treatment of obesity.

## Abbreviations

BAT: brown adipose tissue
BMI: body mass index
BMP8b: bone morphogenetic protein-8b
cAMP: cyclic adenosine monophosphate
C/EBP-α: CCAAT-enhancer-binding protein-α
HFD: high fat diet
GLP-1: glucagon-like peptide-1
GPDH: glycerol-3-phosphate dehydrogenase
Muc2: mucin 2 gene
NF-κB: nuclear factor-κB
PGC1-α: PPARγ co-activator 1-α
PKA: protein kinase A
PPARα: peroxisome proliferator-activated receptor-α
PPARγ: peroxisome proliferator-activated receptor-γ
PRDM-16: positive regulatory domain containing 16
PR: positive regulatory
Reg3g: regenerating islet-derived protein 3γ
SIRT-1: sirtuin-1
STAT-3: signal transducer and activator of transcription-3
T2DM: Type 2 diabetes mellitus
TRPV1: transient receptor potential vanilloid 1
UCP-1: uncoupling protein 1
WAT: white adipose tissue
WHO: World Health Organization
